# Pro-Inflammatory Priming of the Brain: The Underlying Cause of Parkinson’s Disease

**DOI:** 10.3390/ijms24097949

**Published:** 2023-04-27

**Authors:** Ana Catarina Martins, Illyane Sofia Lima, Ana Catarina Pêgo, Inês Sá Pereira, Gracelino Martins, Antonino Kapitão, Raffaella Gozzelino

**Affiliations:** NOVA Medical School Research, NOVA University of Lisbon, 1169-056 Lisbon, Portugal

**Keywords:** iron metabolism, immunity, infection, neuroinflammation, Parkinson’s disease

## Abstract

Parkinson’s disease (PD) is a multifactorial neurodegenerative pathology characterized by the progressive loss of dopaminergic neurons in the substantia nigra of the brain. Aging is considered the main risk factor for the development of idiopathic PD. However, immunity and inflammation play a crucial role in the pathogenesis of this disorder. In mice, we showed that pro-inflammatory priming of the brain sensitizes to severe PD development, regardless of animal age. Age-related sub-acute inflammation, as well as the activation of the immune response upon exposure to harmful stimuli, enhances PD manifestations. The severity of PD is influenced by the engagement of host resistance mechanisms against infection based on the removal of iron (Fe) from the circulation. The sequestration of Fe by immune cells prevents pathogens from proliferating. However, it leads to the formation of a Fe-loaded circulating compartment. When entering the brain through a compromised blood-brain barrier, Fe-loaded immune cells contribute to enhancing neuroinflammation and brain Fe overload. Thus, pro-inflammatory priming of the brain exacerbates neuronal damage and represents a risk factor for the development of severe PD symptoms. Further investigations are now required to better understand whether therapeutic interventions inhibiting this phenomenon might protect against PD.

## 1. Introduction

Parkinson’s disease (PD) is a progressive and multifactorial neurodegenerative disorder. PD etiology is still poorly understood. Aging is considered the main risk factor. However, genetic and environmental influences were also correlated to PD onset [1]. The lack of direct evidence causing PD hindered the development of specific therapies. There is no cure for this pathology. Available drugs might alleviate PD symptoms and retard its progression but do not prevent disease manifestations [2,3].

PD is caused by the loss of dopaminergic neurons (DNs) in the substantia nigra of the brain [4,5]. Its prevalence doubled since 2000. In 2019, approximately 8.5 million people were diagnosed with PD. This represents 1% of the entire population over 60 years and 5% of individuals over 85 years of age [6]. PD is associated with the development of motor and non-motor symptoms. Clinical signs range from tremors at rest, bradykinesia, dystonia and gait disturbances to mood and sleep disorders [7]. Cognitive impairment affects 30–40% of PD patients, who present an increased risk of developing severe dementia [8]. PD is also associated with the development of gastrointestinal (GI) manifestations, frequently reported before the appearance of motor symptoms [9,10,11]. The direct communication between the gut and the brain is provided by the autonomous nervous system [12,13]. Truncal vagotomy was shown to diminish the risk for PD development [14]. Hence, the gut started to be investigated as a potential target for therapeutic intervention against PD. This was also encouraged by the existence of a retrograde transport of α-synuclein (α-syn) to the brain [15], where it forms aggregates known as Lewy bodies (LBs). LBs are a hallmark of PD [16,17]. Thus, peripheral events were shown to contribute to the development of this pathology. Pro-inflammatory cytokines were detected in the blood of PD patients [18], while mice lacking genes encoding for pro-inflammatory mediators developed a less severe phenotype [19,20,21]. Peripheral immune cells were also found in the post-mortem brains of PD patients [22] and associated with enhanced neuroinflammation and PD progression [23]. In mice, the genetic depletion of a specific immune cell population, namely Helper CD4 T cells, was found to protect against PD severity [24].

Among the features that characterize PD, the increase in brain iron (Fe) stands out [25]. The causes that lead Fe to accumulate in the brain are still poorly understood. However, Fe was shown to contribute to neuronal damage [26]. Clinical trials with Fe chelation therapy produced results that are far from satisfactory [27]. Our study demonstrates that peripheral events play a crucial role in mediating Fe-driven DNs loss. Hence, a different therapeutical schedule of deferiprone could be tested against PD by administering the Fe chelator before the appearance of neurodegenerative symptoms. Aging is associated with the formation of circulating Fe-loaded cells [28,29,30], a phenomenon also occurring in response to harmful stimuli that activate the immune system [31,32,33,34]. The sequestration of Fe by immune cells prevents pathogens from accessing one of the nutrients that is essential for their proliferation. By withholding Fe, immune cells become pro-inflammatory, an effect that might damage sensitive organs [35], like the brain [36,37]. The infiltration of Fe-loaded immune cells into the brain through a compromised blood-brain barrier (BBB) enhances neuroinflammation, regardless of the mice’s age.

Thus, our investigation might set the basis for circulating Fe-loaded immune cells to be used as a prognostic marker for PD severity and, possibly, for other neurodegenerative diseases characterized by brain Fe accumulation [32,38,39,40,41,42]. The relevance of our findings could also aid the development of future therapeutic interventions against PD. By targeting the periphery and preventing the damage induced by the pro-inflammatory priming to the brain, those drugs might protect against severe PD manifestations without the need to cross the BBB.

## 2. Results

### 2.1. Aging Increases the Severity of Parkinson’s Disease

Aging is considered the main risk factor for neurodegenerative diseases like PD [43,44]. Accordingly, aged mice were expected to develop a more severe phenotype, in terms of locomotor dysfunction, in response to the induction of pharmacologic PD. PD was triggered by MPTP, a neurotoxin precursor that, in the brain, is metabolized into the active compound by glial cells. MPTP is used to mimic the last stage of PD, i.e., DNs loss, given its ability to target specifically DNs. PD was induced in C57BL/6 mice aged between 8–12 weeks and referred to as “young.” Disease severity was compared to animals aged 52–60 weeks and referred to as “old.” Mice were monitored daily, for 30 days, after MPTP administration. When compared to standard protocols [45], mice received sub-acute doses of MPTP to better appreciate differences between the conditions that were tested.

**Figure 1:**
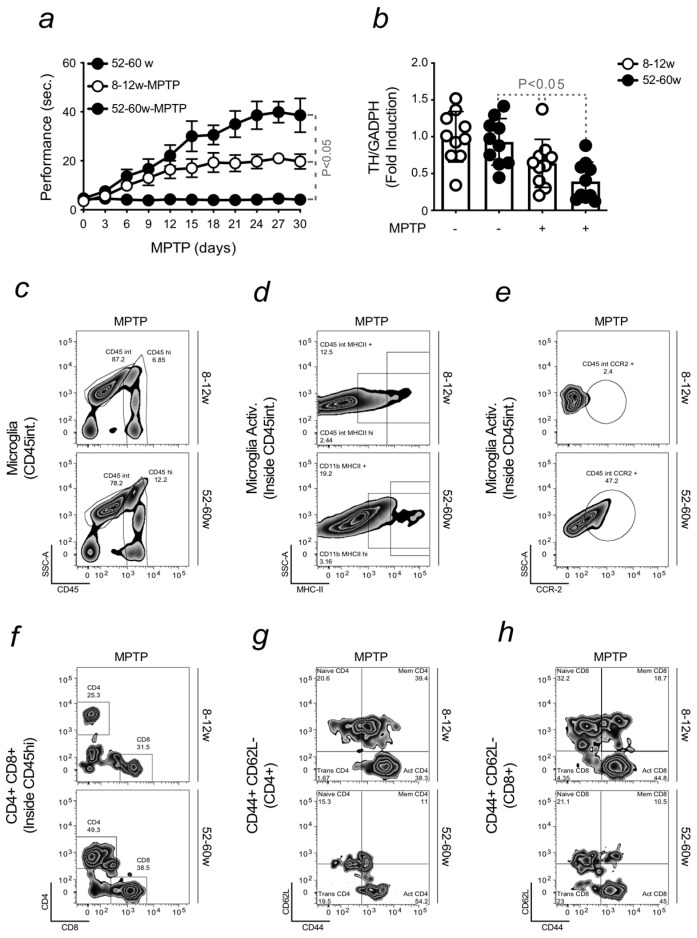
**Aging increases the severity of Parkinson’s disease**. All mice were of a C57BL/6 background (**a**) 8–12 weeks old (young) and 52–60 weeks (old) mice were intoxicated with sub-acute doses of MPTP (15 mg/kg, i.p., 3 injections 2 h apart). Non-treated 52–60 weeks (old) mice were used as controls. Motor dysfunction was evaluated by assessing the time of performance on a pole test. Mice were monitored for 30 days after MPTP administration. (**b**) Tyrosine Hydroxylase (*TH*) mRNA expression, quantified by qRT-PCR upon isolation of the substantia nigra of the brain of mice as in (**a**). The results were normalized to *GADPH*, used as a housekeeping gene, and expressed as mean ± SD (*n* = 10 mice per group). Statistical analysis was performed by applying the one-way ANOVA test. (**c**) Representative flow cytometry dot plot showing the gating strategy of freshly isolated infiltrated leukocytes (CD45hi) and microglia (CD45int) from the brain of PD-induced young and old mice. A standardized gating strategy was used as follows: first, we eliminated doublets and clumps by Forward Scatter (FSC) FSC-Height vs. FSC-Area, from a density plot. Then according to their FSC/Side Scatter (FSC/SCC) profile, debris were eliminated, and leukocytes were gated. Total live leukocytes were identified based on the exclusion of PI-positive marker (viable cells are impermeable to PI). Using a combination of CD11b and CD45 markers, it was possible to distinguish microglia from peripheral immune cells according to their expression of CD45. (**d**,**e**) Activated microglia (CD45int MHCII+ and CD45int CCR2+), within contour plots, of PD-induced young and old mice, assessed by the expression of inflammatory markers, as the Major Histocompatibility II (MHCII) and the recruiting chemokine CCR2. (**f**–**h**) T cells population were identified by combining SSC-A and TCRβ markers. Gating strategy and activation status of freshly isolated infiltrated Helper (CD4+) and Cytotoxic (CD8+) T cells from PD-induced young and old mice, representing from the upper left quadrant, clockwise, naïve (CD44-CD62L+), memory (CD44+CD62L+) and activated cells (CD44+CD62L-). Infiltrated lymphocytes (CD45hi) cells were gated as in (**c**) and further identified using a TCRβ marker. Those cells were subsequently subdivided into CD4+ and CD8+ populations.

The results demonstrated that, contrary to young mice, the induction of PD in aged animals was associated with the development of severe locomotor dysfunction. Non-treated old mice were used as controls (Figure 1a). Motor symptoms were evaluated using the pole test, an assessment of motor coordination that measures the performance of mice placed upside-down on a vertical pole [46]. Balance impairment was associated with DNs loss. This was measured by qRT-PCR, quantifying the expression of tyrosine hydroxylase (*TH*), the enzyme responsible for dopamine synthesis, in samples isolated from the substantia nigra. A lower expression of *TH* was found in old mice induced with MPTP when compared to young animals developing PD or age-matched controls. It is important to note that these results also validate the use of MPTP as a pharmacologic PD model, in agreement with the scientific literature [47]. No significant differences in DNs loss were observed in young mice, induced or not with PD. This result is possibly justified by milder symptoms developed by young animals in response to MPTP (Figure 1b). However, we confirmed that aging increases PD severity. This finding was also corroborated by the pronounced neuroinflammatory phenotype, as shown by the higher activation of microglia in old mice that were exposed to MPTP (Figure 1c–e). The quantification of pro-inflammatory markers, like MHCII and CCR2, confirmed the results obtained (Supplementary Figure S1a,b). Neuroinflammation is possibly enhanced by the infiltration of peripheral immune cells into the brain of old mice exposed to MPTP. An increase in CD4 Helper and CD8 Cytotoxic T cells was detected in the brain of PD-induced aged animals (Figure 1f). CD44 was used as a marker of activation and the expression of CD62L as a naïve cell phenotype, which is lost during cell differentiation. CD44 expression was higher in both immune cell populations in the same mice (Figure 1g,h). The correspondent plot quantification confirmed increased neuroinflammation developed by old mice in relation to young animals upon PD induction (Supplementary Figure S1c–f).

Overall, these data confirmed that aging is associated with the development of a more severe PD phenotype in response to MPTP.

### 2.2. Aging-Associated Inflammation Promotes the Formation of Fe-Loaded Inflammatory Cells

**Figure 2:**
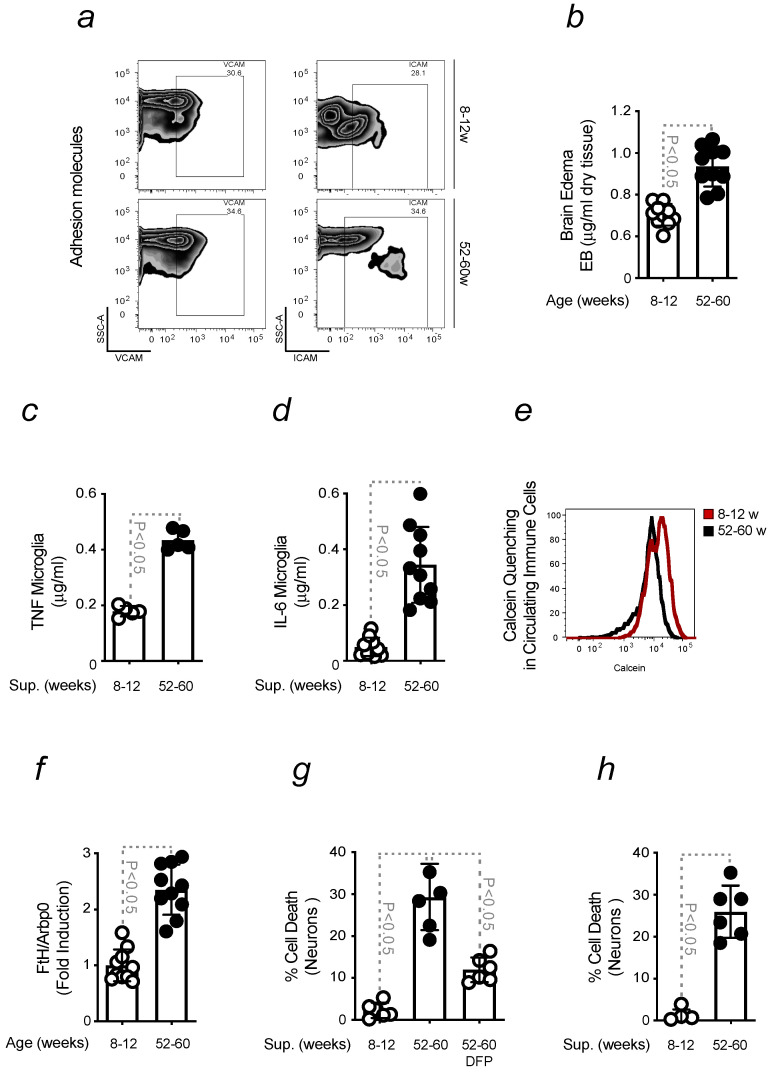
**Aging-associated inflammation promotes the generation of inflammatory Fe-loaded cells**. All mice were of a C57BL/6 background (**a**) Gating strategy for the detection of adhesion molecules, VCAM and ICAM, from blood-brain barrier endothelial cells (CD31+), freshly isolated from the brain of non-treated 8–12 weeks old (young) and 52–60 weeks (old) mice. (**b**) BBB permeability, measured by the extravasation of Evans Blue dye into the brain and referred to as Brain Edema, in young vs. old mice, as in (**a**). (**c**) TNF and (**d**) IL–6 levels, measured by ELISA, in the supernatant collected from microglia, upon stimulation with sera collected from mice as in (**a**). (**e**) Calcein median fluorescence intensity, measured by flow cytometry, in immune cells isolated from sera collected from mice as in (**a**). (**f**) mRNA expression of *FtH*, measured by qRT–PCR in cells as in (**e**) collected from mice as in (**a**), which results were normalized to *ArbP0* (**g**) Neuronal viability, assessed by Crystal Violet staining, upon stimulation with sera collected from young and old mice, pre-treated or not with deferiprone (DFP). (**h**) Neuronal viability was assessed by Crystal Violet staining in response to stimulation with supernatants collected from microglia previously exposed to sera from young and old mice. The results were expressed as mean ± SD (*n* = 7–10 mice per group). The Student’s *t*-test was applied to define statistical differences.

Comparative analyses were carried out to better understand the causes underlying an increased sensitivity of old mice to PD when compared to young animals. We recently demonstrated that aged mice develop a neuroinflammatory phenotype, a physiological process also occurring during healthy aging [31]. Increased permeability of the BBB was shown to contribute to this phenomenon. In the absence of PD, a higher expression of adhesion molecules, ICAM and VCAM, was observed by flow cytometry in the brain of old mice when compared to young animals (Figure 2a). The correspondent plot quantification confirmed the data obtained (Supplementary Figure S2a,b). An impaired BBB integrity was found in aged animals. BBB permeability was measured by the extravasation of Evans Blue dye into the brain when administered intravenously (Figure 2b). A compromised BBB favored the infiltration of immune cells into the brain. We performed in vitro experiments to test the ability of peripheral cells to switch microglia towards a pro-inflammatory phenotype. Isolated primary microglia were stimulated with sera collected from young and old animals, and the release of pro-inflammatory cytokines was measured by ELISA. Higher levels of TNF and IL-6 were detected in response to sera from old mice (Figure 2c,d). To understand the reason for this phenomenon, we assessed its correlation with the generation of Fe-loaded cells [31]. Calcein quenching is an indirect method to measure Fe overload. However, it indicates the intracellular accumulation of Fe in circulating immune cells. Differences in calcein quenching were observed when comparing young and old mice (Figure 2e). The notion that peripheral immune cells were Fe-loaded was confirmed by quantifying the expression of *FtH* by qRT-PCR. An increased *FtH* was found in circulating immune cells when isolated from aged mice (Figure 2f). To mimic the damage induced by Fe-loaded cells to neurons, we exposed primary DNs in vitro to sera collected from young and old mice. An increased neuronal death was observed when neurons were treated with sera collected from aged animals. Pre-treatment with Fe chelator, deferiprone, was found to prevent DNs loss (Figure 2g). Similar toxicity was observed when DNs were stimulated with supernatant collected from microglia, which were previously exposed to sera collected from old mice (Figure 2h). The treatment turned microglia pro-inflammatory and capable of triggering neuronal damage.

Overall, these data indicate that PD results from the accumulation of peripheral events, which critically contribute to the pathogenesis of this disease.

### 2.3. Brain Fe Accumulation and Inflammation Sensitize to an Enhanced PD Severity

**Figure 3:**
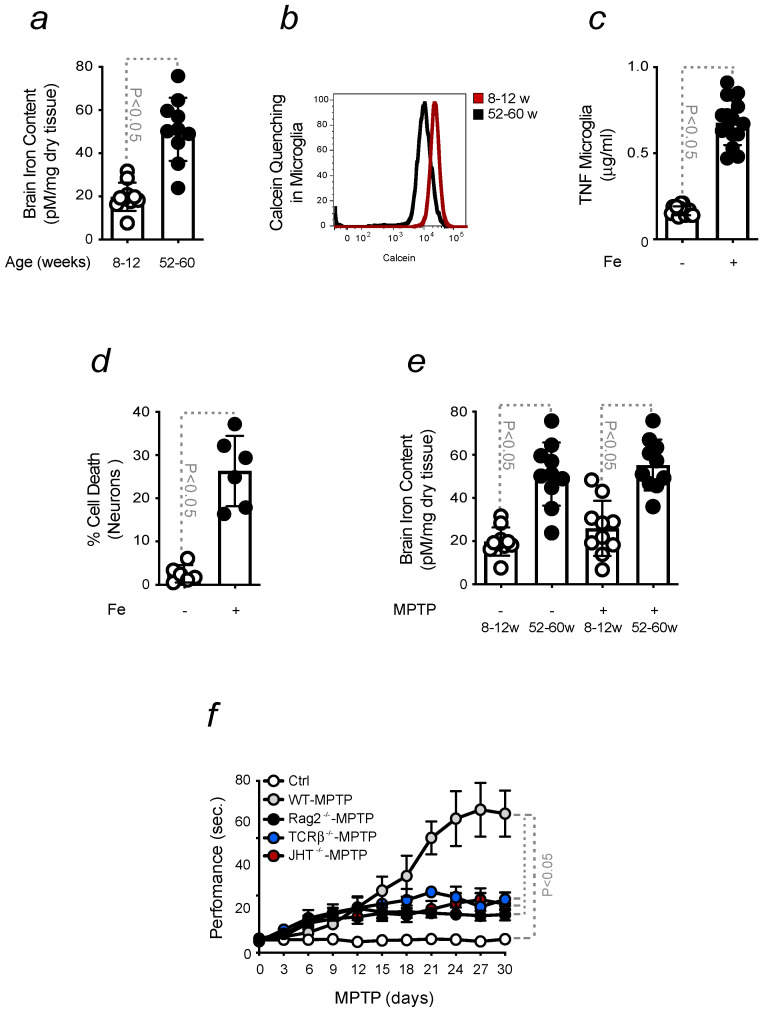
**Brain Fe and inflammation sensitize towards an enhanced PD severity**. All mice were in C57BL/6 background (**a**) Intracellular Fe content measured in the brain of 8–12 weeks old (young) and 52–60 weeks (old) mice. (**b**) Calcein median fluorescence intensity, measured by flow cytometry, in immune cells isolated from sera collected from mice as in a). (**c**) TNF levels, measured by ELISA, in the supernatant collected from microglia, upon stimulation with Fe (150 μM). (**d**) Neuronal viability, assessed by Crystal Violet staining in response to Fe stimulation (150 μM). (**e**) Brain Fe content was measured in young mice, exposed or not to MPTP. All results presented so far were expressed as mean ± SD (*n* = 6–10 mice per group). (**f**) Locomotor dysfunction of young wild-type, Rag-2-deficient, TCRβ-deficient or JHT-deficient mice, aged between 8–10 weeks and in C57BL/6 background, intoxicated with high doses of MPTP (20 mg/kg, i.p., 4 injections 2 h apart). Motor dysfunction was evaluated by assessing the time of performance on a pole test. Mice were monitored for 30 days after MPTP administration. The results are expressed as mean ± SD (*n* = 10 mice per group). Statistical analysis was performed by applying the one-way ANOVA test.

Aging is associated with an increased level of brain Fe (Figure 3a). Microglia are considered the reservoir of Fe in the brain. Their ability to accumulate Fe was confirmed by calcein quenching, evaluated in microglia isolated from young and old animals (Figure 3b). The pro-oxidant nature of Fe is capable of switching microglia towards a pro-inflammatory phenotype [48]. This was also demonstrated by the release of pro-inflammatory cytokines when these cells were exposed to Fe. An increased level of TNF was assessed in the supernatant of primary microglia treated with Fe (Figure 3c). Increased cell death was also observed when DNs neurons were exposed to Fe (Figure 3d). While the neuroinflammation, developed by old mice, is associated with an increased brain Fe accumulation, no differences were found in young and old animals upon PD induction (Figure 3e). This indicated that Fe accumulation in the brain occurs before PD onset and is not induced by disease progression. Hence, we showed that the activation of the immune system primes the brain to Fe overload and neurodegeneration. The contribution of the immune system to PD development was confirmed by using mice deprived of adaptive immunity. Rag-2-deficient, TCRβ-deficient or JHT-deficient mice, depleted of T and/or mature B cells, were found to be more resistant to PD induction in relation to aged-matched wild-type animals (Figure 3f).

Overall, these data indicate that the infiltration of peripheral immune cells into the brain is key to enhancing PD severity.

### 2.4. Infection-Driven Pro-Inflammatory Priming to the Brain Promotes Neuroinflammation

**Figure 4:**
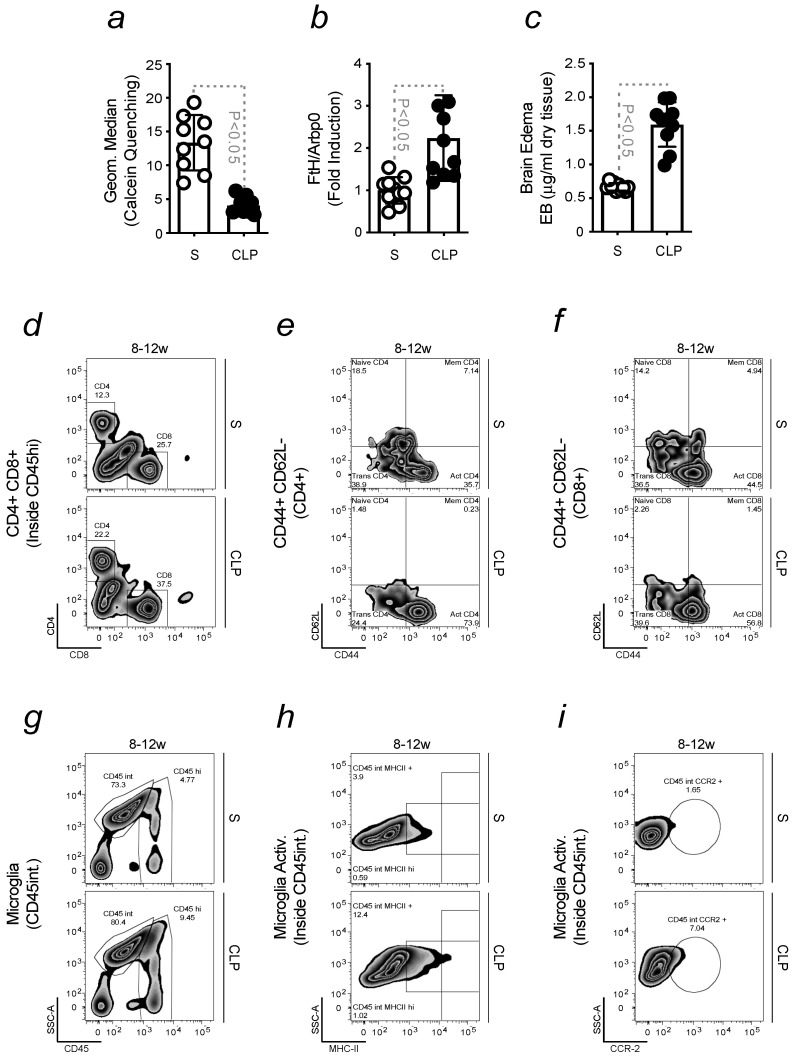
**Infection-driven pro-inflammatory priming to the brain promotes neuroinflammation**. All mice were of a C57BL/6 background (**a**) Calcein median fluorescence intensity, measured by flow cytometry, in leukocytes isolated from sera collected from mice subjected to sham surgery (S) or cecum ligation and puncture (CLP). (**b**) mRNA expression of *FtH*, measured by qRT-PCR in cells collected from mice as in (**a**), which results were normalized to *ArbP0*. (**c**) BBB permeability, measured by the extravasation of Evans Blue dye into the brain and referred to as Brain Edema, in young vs. old mice, as in (**a**). So far, the results were expressed as mean ± SD (*n* = 10 mice per group). The Student’s *t*-test was applied to define statistical differences. (**d**) Gating strategy and activation status, detailed in the legend of Figure 1, of freshly isolated infiltrated Helper (CD4+) and Cytotoxic (CD8+) T cells from mice as in (**a**), and their activation, assessed in (**e**) CD4 and (**f**) CD8 by the activation marker CD44. From the upper left quadrant, clockwise, plots identify naïve, memory and activated cells. (**g**) Gating strategy to obtain freshly isolated infiltrated microglia (CD45int), within contour plots, from mice as in (**a**). (**h**) Activated microglia (CD45int MHCII+) and (**i**) (CD45int CCR2+), within contour plots, in mice as in (**a**).

Considering the contribution of peripheral immune cells in enhancing PD severity, we assessed whether the activation of the immune system, in response to the infection, could mimic the phenotype observed in the brain of aged mice. Young animals were subjected to mild cecum ligation and puncture (CLP), and the results obtained were compared to mice exposed to sham surgery (S). The generation of Fe-loaded immune cells was observed in CLP-induced mice, not in S mice. The accumulation of Fe was assessed by calcein quenching (Figure 4a), and the expression of *FtH* in immune cells was measured by qRT-PCR (Figure 4b). We previously demonstrated that the inflammatory status associated with aging correlates to increased permeability of the BBB. Its integrity was disrupted in mice subjected to CLP (Figure 4c). The increased expression of *VCAM* in CLP-induced mice was quantified by qRT-PCR and was shown to confirm previous findings (Supplementary Figure S3a). In agreement, an enhanced infiltration of CD4 Helper T and CD8 Cytotoxic T cells was found in mice upon CLP (Figure 4d). Immune cell count and activation were increased in CPL-induced mice, as shown by the expression of the CD44 activation marker on subset populations (Figure 4e,f; Supplementary Figure S3b–e). Then, we assessed whether peripheral infections sensitize the brain to neuroinflammation by evaluating the number (Figure 4g) and activation of microglia by flow cytometry. MHCII and CCR2 were used as activation markers, and their expression was quantified (Figure 4h,i; Supplementary Figure S3f–h). A time course was performed to assess whether the infiltration of immune cells into the brain upon CLP was an early symptom. The results obtained demonstrated that this is the case. Peripheral immune cells were detected in the brain a few days after CLP, as well as the activation of microglia (Supplementary Figure S4). This finding further confirms that the immune response elicited upon infection is a risk factor for the development of a neuroinflammatory phenotype.

**Figure 5:**
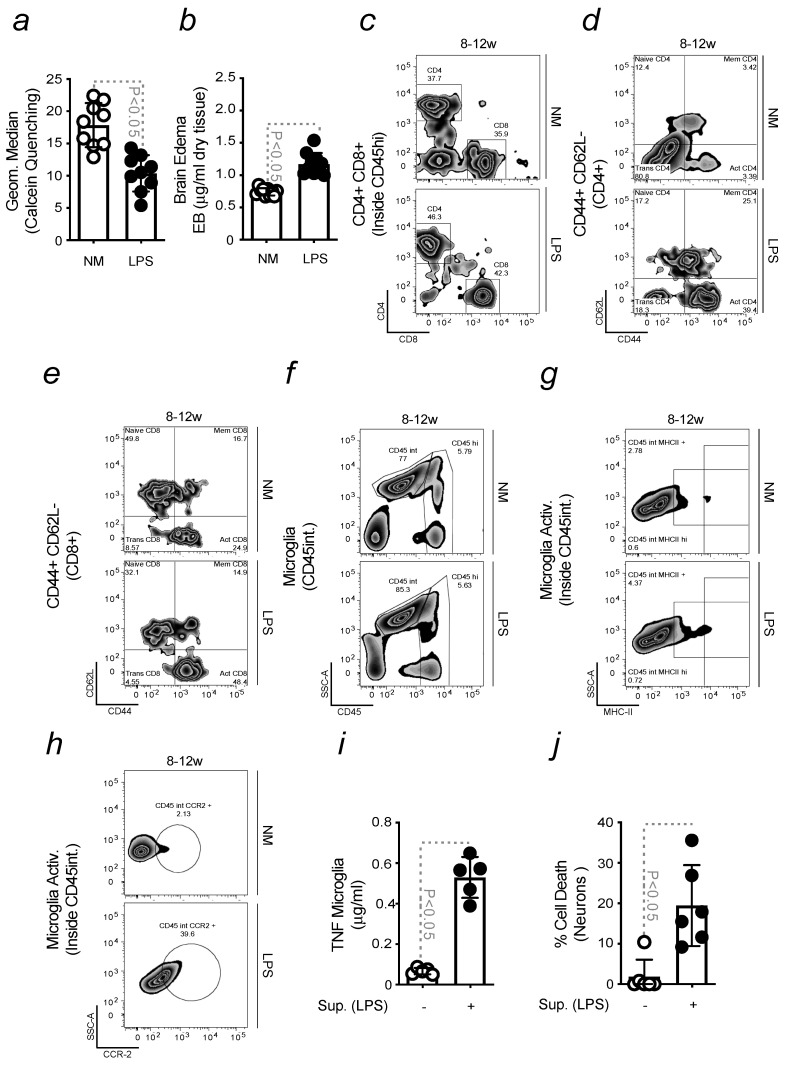
**Infection-driven pro-inflammatory priming to the brain promotes neuroinflammation.** All mice were of a C57BL/6 background (**a**) Calcein median fluorescence intensity, measured by flow cytometry, in leukocytes isolated from sera collected from mice exposed or not to LPS (**b**) BBB permeability, measured by the extravasation of Evans Blue dye into the brain and referred as Brain Edema, in young vs. old mice, as in (**a**). So far, the results were expressed as mean ± SD (*n* = 10 mice per group). The Student’s *t*-test was applied to define statistical differences. (**c**) Gating strategy, detailed in the legend of Figure 1, of freshly isolated infiltrated Helper (CD4+) and Cytotoxic (CD8+) T cells from mice as in (**a**), and their activation, assessed in (**d**) CD4 and (**e**) CD8 by the activation marker CD44. From the upper left quadrant, clockwise, plots identify naïve, memory and activated cells. (**f**) Gating strategy to obtain freshly isolated microglia (CD45int), within contour plots, from mice as in (**a**). (**g**) Activated microglia (CD45int MHCII+) and (**h**) (CD45int CCR2+), within contour plots, in mice as in (**a**). (**i**) TNF levels, measured by ELISA, in the supernatant collected from microglia, upon stimulation with sera collected from mice as in (**a**). (**j**) Neuronal viability, assessed by Crystal Violet staining, in response to stimulation with supernatants collected from microglia previously exposed to sera from mice as in (**a**).

Similar data were obtained when mice were exposed to LPS (Figure 5a–h; Supplementary Figure S5). The contribution of peripheral immune cells to microglia activation and neurodegeneration was also demonstrated in vitro. When primary microglia were exposed to sera collected from LPS-induced mice, the release of TNF in the supernatant increased (Figure 5i). When this supernatant was used to stimulate isolated DNs, we observed a reduction in cell viability (Figure 5j).

### 2.5. Pro-Inflammatory Priming to the Brain Enhances PD Severity

**Figure 6:**
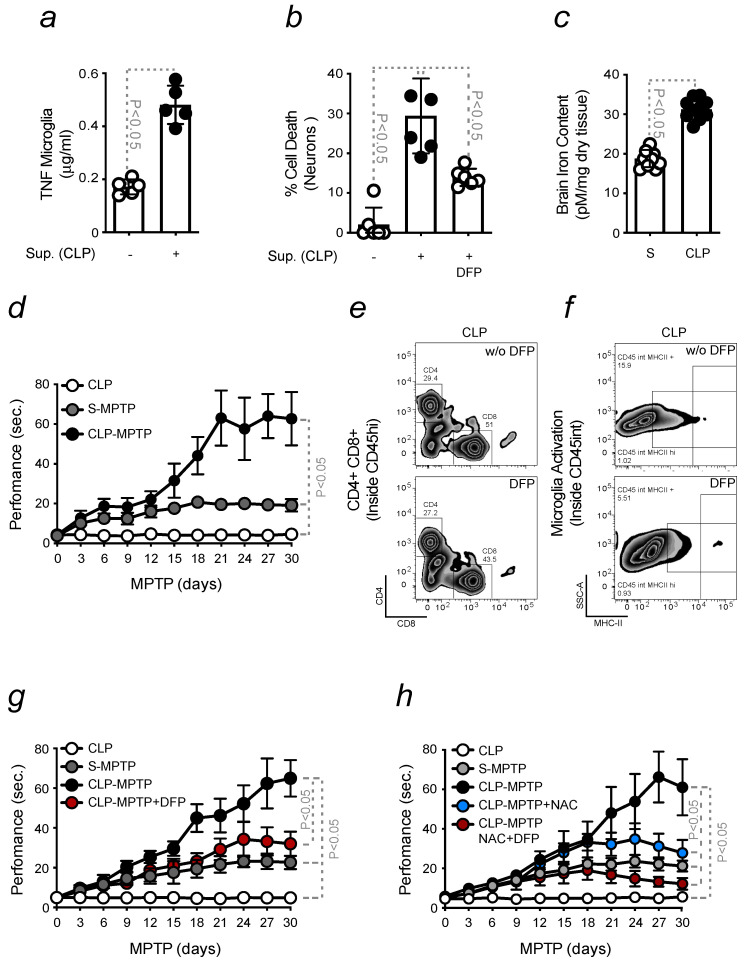
**Pro-inflammatory priming to the brain enhances PD severity**. All mice were of a C57BL/6 background and aged 8–12 weeks (**a**) TNF levels, measured by ELISA, in the supernatant collected from microglia, upon stimulation with sera collected from mice subjected or not to CLP. (**b**) Neuronal viability, assessed by Crystal Violet staining, in response to stimulation as in a) receiving or not deferiprone (DFP) treatment. (**c**) Intracellular Fe content measured in the brain of Sham (S) or CLP mice. So far, the results were expressed as mean ± SD (*n* = 10 mice per group). The Student’s *t*-test was applied to define statistical differences. (**d**) Locomotor dysfunction measured in C57BL/6 mice, induced or not with CLP and intoxicated with MPTP (15 mg/kg, i.p., 3 injections 2 h apart). Motor dysfunction was evaluated by assessing the time of performance on a pole test. Mice were monitored for 30 days after MPTP administration. (**e**) Gating strategy, detailed in the legend of Figure 1, of freshly isolated infiltrated Helper (CD4+) and Cytotoxic (CD8+) T cells from mice exposed to CLP and therapeutically treated or not with DFP. (**f**) Gating strategy to obtain freshly isolated activated microglia (CD45int MHCII+), within contour plots, in mice as in e). (**g**) Locomotor dysfunction in mice, induced or not with CLP, intoxicated with MPTP (15 mg/kg, i.p., 3 injections 2 h apart) and treated or not with deferiprone (DFP). (**h**) Locomotor dysfunction in mice, induced or not with CLP, intoxicated with MPTP (15 mg/kg, i.p., 3 injections 2 h apart) and treated or not with the combination of DFP and NAC. The results are expressed as mean ± SD (*n* = 10 mice per group). Statistical analysis was performed by applying the one-way ANOVA test.

As in previous experiments, we exposed microglia to sera collected from CLP-induced mice, and we assessed if those cells became pro-inflammatory. An increase in the release of TNF was measured in the supernatant of microglia stimulated with sera from CLP-induced mice (Figure 6a). In contact with DNs, the same sera induced neuronal death; cytotoxicity was prevented by pre-treating cells with deferiprone (Figure 6b). Increased BBB permeability was shown by CLP-induced mice. In these animals, peripheral inflammation was associated with brain Fe accumulation (Figure 6c). PD severity was enhanced in mice that underwent CLP before MPTP induction. Locomotor dysfunction significantly increased when compared to S-induced mice. Mice subjected to CLP but not receiving MPTP were used as controls (Figure 6d). These findings revealed that inflammation primes the brain and sensitizes it to severe PD. Since this phenomenon is characterized by neuroinflammation and Fe accumulation, we assessed whether exposing mice to Fe chelation therapy upon infection conferred protection to the brain. Reduced infiltration of activated peripheral leukocytes was found in the brain of CLP-induced mice, which were therapeutically treated with deferiprone (Figure 6e; Supplementary Figure S6a,b). A reduced microglia activation was also observed, as assessed by flow cytometry, quantifying the expression of the MHCII activation marker (Figure 6f; Supplementary Figure S6c). When mice received deferiprone, starting five days after CLP, a reduced PD severity was observed in relation to PD mice subjected to CLP but not receiving Fe chelation. CLP mice not exposed to MPTP were used as controls (Figure 6g). As Fe is a pro-oxidant, we assessed whether the combination of an antioxidant and Fe chelation therapy prevents CLP mice from developing severe locomotor dysfunction in response to MPTP. Our findings showed that this was the case. The administration of deferiprone and N-acetylcysteine (NAC) significantly reduced PD severity in mice previously subjected to CLP. Beneficial effects were also observed by exposing those mice to NAC alone, although the locomotor dysfunction did not decrease to the same extent as when mice were receiving a combined therapy (Figure 6h).

Overall, these data demonstrated that while a pro-inflammatory priming of the brain enhanced PD severity, a combined treatment of Fe chelator and antioxidants protects mice against the development of severe PD symptoms. This indicates the existence of cross-talk between iron metabolism and inflammation, which ultimately affects the brain.

## 3. Discussion

Aging is the main risk factor for the development and progression of idiopathic PD [49,50]. There is no animal model that fully resembles the pathology [51,52]. However, mice are widely used to mimic multiple aspects of human PD [53]. PD was induced by MPTP, a neurotoxin precursor capable of crossing the BBB due to its lipophilic nature. In the brain, MPTP is metabolized by glial cells into its active form, MPP+. This neurotoxic metabolite is highly toxic to DNs, causing mitochondrial dysfunction and cell death once taken up by dopaminergic transporters [54]. Its affinity for DNs leads MPP+ to reproduce the final phases of PD, i.e., DNs loss [55].

During aging, physiological changes sensitize the body to progressive tissue degeneration, which is often associated with organ failure and disease onset. In agreement, severe symptoms were observed when PD was induced in aged mice when compared to young animals. This effect occurred even upon the administration of sub-acute doses of MPTP, indicating that aging predisposes mice to develop a more pronounced phenotype. Impaired motor coordination was associated with DNs loss. Different factors contribute to neurodegeneration, among which is the activation of microglia. This is enhanced by the infiltration of peripheral immune cells, like Helper CD4 and Cytotoxic CD8 T cells, into the brain, through an impaired BBB. These events characterize age-related neuroinflammation and, as such, constitute a risk factor for PD development (Figure 1). No further increase in BBB permeability was observed upon PD induction, proving that this phenomenon physiologically occurs with advancing age. The contribution of a low-grade neuroinflammatory phenotype, developed during aging, to neuronal damage, was confirmed in vitro. Higher levels of pro-inflammatory cytokines were released when microglia were stimulated with sera collected from old mice. This effect was attributed to the ability of Fe to switch microglia phenotype towards a pro-inflammatory state upon their interaction with peripheral immune cells. Immune cells presented an increased level of intracellular Fe, as assessed by calcein quenching and FtH expression. Fe accumulation was not observed in the same immune cells isolated from young mice. Increased neuronal cytotoxicity was detected upon stimulation with the same sera, an effect prevented by prior exposure to deferiprone. When neurons were exposed to the supernatant collected from the aforementioned microglia, we observed an increase in neuronal loss (Figure 2). This indicated that the pro-inflammatory action of peripheral immune cells and microglia might damage neurons.

Higher levels of brain Fe are associated with aging and microglia dysfunction [26,56,57]. Senescent microglia become dystrophic, accumulate Fe [58] and increase ferritin expression. Dystrophic microglia were found in Lewy bodies in the substantia nigra, in post-mortem samples of PD patients [58,59]. Enhanced calcein quenching was observed in microglia isolated from old mice but not in those isolated from young animals. The ability of Fe to turn microglia pro-inflammatory was investigated in primary cells. In response to Fe, isolated microglia release pro-inflammatory cytokines, such as TNF. Neurons are also sensitive to Fe stimulation, as observed by the increased neuronal death measured upon treatment (Figure 3). Hence, the disruption of Fe homeostasis in the brain strongly contributes to neuroinflammation and sensitizes disease onset, as previously reported [60]. Fe is highly abundant in the brain, where it is involved in many biological functions [32,33,41,42]. Its ability to undergo oxidation and reduction reactions, exchanging electrons with different substrates, turns it potentially toxic. By participating in the Fenton chemistry, Fe generates highly reactive oxygen species (ROS), which induce oxidative stress and tissue damage, as shown to occur also in the aging brain [60,61,62]. Our data found no increase in the accumulation of Fe in mice exposed or not to MPTP when comparing young vs. old animals (Figure 3). This data confirmed that increased Fe levels occur throughout the lifetime [31,32,63] and are not detected prior to disease onset [31,32,40,41,42]. Sequestered in peripheral immune cells, Fe circulates and enters the brain. The absence of adaptive immunity was shown to benefit PD, reducing its severity. When PD was induced by exposing TCRβ-deficient, Rag-2-deficient, or JHT-deficient mice, depleted of T and/or mature B cells, respectively, to high doses of MPTP, the resulting locomotor dysfunction was not as severe as in aged-matched wild-type animals. Deficient mice were more resistant to PD. This indicates that the immune system primes the brain to an increased disease severity (Figure 3), as recently demonstrated [31]. We showed that a sequence of events is necessary to induce neurodegeneration. To prove this statement, we exposed young mice to an infection-driven pro-inflammatory priming and assessed its contribution to the appearance of a neuroinflammatory phenotype. Our aim was to confirm that pro-inflammatory priming is a risk factor for PD, regardless of mice’s age. During life, we are continuously exposed to different challenges, which cause inflammation and impact sensitive organs like the brain [35,64,65]. Upon infection, immune cells withhold Fe to restrict pathogen proliferation [36]. However, the accumulation of Fe within these cells, confirmed by calcein quenching and FtH expression, turns them pro-inflammatory [31,32]. We detected Fe-loaded immune cells in young mice exposed to mild CLP or sub-acute doses of LPS, which infiltrate the brain due to an increased BBB permeability. Consequently, microglia turn pro-inflammatory and enhance neuroinflammation (Figure 4 and Figure 5). In vitro experiments were also conducted to assess the capacity of sera, collected from mice exposed to sub-acute doses of LPS, to activate microglia and induce the release of pro-inflammatory cytokines, like TNF. The supernatant of stimulated microglia was used to treat neurons, showing it causes neuronal death (Figure 5). Similar data were observed by stimulating microglia or neurons with sera collected from CLP-induced mice, as assessed by the release of pro-inflammatory cytokines like TNF or DNs loss, respectively. This latter was inhibited by deferiprone, indicating the role of Fe in promoting neurodegeneration. To further demonstrate that the pro-inflammatory priming to the brain exacerbates PD severity, we exposed mice to CLP prior to MPTP intoxication and observed an increase in locomotor dysfunction. The protective effect of deferiprone against CLP-induced neuroinflammation further confirmed the involvement of Fe. The therapeutic administration of the Fe chelator administered a few days after CLP, prevented PD severity. Although Fe is necessary to elicit an immune response, its chelation is often required to prevent Fe-driven organ damage [66]. Since Fe is also a pro-oxidant, we assessed whether the combination of an antioxidant compound, NAC, and deferiprone protected against PD severity. The synergistic effect of this treatment confirmed that PD symptoms can be ameliorated by acting at a peripheral level. This notion is also in agreement with the administration routes of our therapy. Hence, our data clearly showed that, regardless of the mice’s age, the pro-inflammatory priming to the brain sensitizes to neuroinflammation and Fe accumulation, both being key players in PD. The findings described in this study are consistent with the observation that certain infections induce neurological signs in patients, even if the brain is not directly affected [67,68,69].

## 4. Materials and Methods

*Mice*

C57BL/6J mice were purchased from the Champalimaud Foundation, Portugal, and immuno-compromised mice Rag2−/−, TCRβ−/− and JHT−/− from Instituto Gulbenkian de Ciência (IGC) Oeiras, Portugal. Animals were bred and maintained under SPF conditions. Experimental protocols were approved by the ethics committees of the institutions above and the Portuguese National Entity (Direcção Geral de Alimentação e Veterinária; 0421/000/000/2018), according to the Portuguese (Decreto-Lei 113/2013) and European (Directive 2010/63/EU) legislations.

All mice were maintained in cages (3–5 animals per cage), undisturbed, in an environmentally controlled room, in terms of temperature and humidity conditions, with a 12 h light/12 h dark cycle, and fed a standard diet and water at libitum. Animal care was taken to ensure that any mice exhibiting signs of suffering or distress were euthanized with CO_2_. This also included animals showing normal conditions of disruption and general ill-health signs, causing difficulty eating or drinking, moderate discomfort, pain or distress. Severe forelimb, hindlimb incoordination and locomotor disabilities constituted end-point criteria to sacrifice the animals used in our experiments. All symptoms were noted, registered, and discussed with the veterinarian of the animal facility.

The hypothermia caused by MPTP administration was prevented by using a heating pad, to which mice were allocated before the procedure. MPTP-induced mice were then transferred into pre-heated cages 2 h after the last injection. Animal cages were changed every 96 h after the procedure, and the mice’s well-being was monitored until experiment day 30.

*Reagents*

Cells (isolated from C57BL/6 mouse brain) were cultured in DMEM (Invitrogen, Waltham, MA, USA), supplemented with 10% FCS, penicillin (20 U/mL), and streptomycin (20 U/mL) (Invitrogen) (37 °C, 95% humidity, 5% CO_2_). N-acetylcysteine (NAC) and deferiprone were purchased from Sigma. M-CSF and GM-CSF were purchased from R&D Systems, UK. Laminin, DMEM/F12, Poly-L-ornithine, PBS, BSA, FBS, HBSS, N2 supplement, used for maintaining DNs in culture were purchased by Invitrogen, while D-(+)-Glucose and trypsin-EDTA from Sigma.

*PD Induction and evaluation of locomotor dysfunction*

PD was pharmacologically induced, according to the protocol described in [45]. Mice were injected with 1-methyl-4-phenyl-1,2,3,6-tetrapyridine (MPTP; Sigma-Aldrich Ref. No. M0896, Darmstadt, Germany) at a dose of 15 mg/kg, i.p. (3 injections 2 h apart) for the sub-acute doses of MPTP and of 20 mg/kg (4 injections, i.p., 2 h apart) for the intoxication with high doses of MPTP. Mice were monitored daily for 30 days. PD was induced 3 weeks after LPS or CLP exposure.

Locomotor dysfunction was assessed by placing mice upside-down on a vertical pole and measuring how many seconds they needed to descend [46]. A default time of performance of 120 s was set in case the severity of induced PD prevented mice from descending the pole.

*Deferiprone treatment in mice*

Mice were injected with deferiprone (DFP, Sigma Aldrich, Ref. No. 379409; Darmstadt, Germany) at a dose of 10 mg/kg (1 injection per day, i.p.). Mice were injected for 15 days, starting 5 days after LPS or CLP injection.

*LPS injection*

Ten μg of LPS were injected intraperitoneally (i.p.) once a day for 5 days to cause a mild inflammatory phenotype. No signs of stress were observed during or after the treatment, according to the health parameters measured daily (weight and temperature). After recovery, i.e., 3 weeks later, PD was induced, and disease progression was monitored for 30 days to then collect blood and organs for further analyses.

*NAC injection*

NAC was dissolved in PBS, adjusted to pH 7.4, and administered to mice (15 mg/kg i.p.) starting 5 days after PD induction and every 12 h thereafter for 15 days.

*Cecum ligation and puncture*

Mice were anesthetized with inhalable anesthetic 1-chloro-2,2,2- trifluoroethyl difluoromethyl ether (isofluorane) supplied at 500–1000 mL/min while maintained in an induction chamber; at 100–200 mL/min they were switched to nosecone to maintain that the animal anesthetized. The abdominal area of the animal was shaved, disinfected, and cleaned with 70% alcohol solution, followed by a betadine disinfectant solution. A small incision (1 cm) was made parallel to the midline. The cecum was exteriorized and ligated immediately distal to the ileocecal valve. In order to perform a low-grade CLP, the lumen was reduced by 50–60%, and the cecum was punctured once with a 23-gauge needle. Fecal content was extruded by applying pressure, and then the cecum was re-inserted into the abdominal cavity. The peritoneal wall was sutured with sterile 3-0 Dafilon sutures (Braun, Kronberg, Germany), and the skin was closed with a surgical staple (Autoclip 9 mm; Becton Dickinson, Franklin Lakes, NJ, USA). During the full procedure, the animal was kept in a temperature stabilizer (37 °C), the eyes were continuously moisturized to avoid blindness, and sterile isotonic fluids (1 mL 0.9% saline) were administrated subcutaneously (s.c.) at the end of the procedure to allow better recovery and fluid resuscitation. At the end of the surgery, the animal was allowed to recover on the temperature stabilizer (37 °C) for an extra 30 min, while antibiotics (Imipenem/Cilastine; Tienam; MSD; 0.5 mg/s.c./animal) were administered 2 h after the procedure and every 12 h during 72 h. Fully awake animals were transferred to standard cages and strictly monitored for health and welfare conditions (activity, behavior, temperature, and weight). The procedure caused little stress to the animal, and the outcome led to a sepsis condition, causing a mild inflammatory phenotype. No death was expected or observed. After recovery, i.e., 3 weeks later, PD was induced, and disease progression was monitored for 30 days to then collect blood and organs for further analyses.

*Blood Brain Barrier permeability*

Mice were injected intravenously (i.v.) with 0.1 mL of 2% Evans Blue (Sigma-Aldrich, Ref. No 2129; Darmstadt, Germany), dissolved in saline solution and were killed 1 h later. Brain samples were harvested, weighed, placed in 2 ml of formamide and left for 48 h at 37 °C to extract the Evans Blue dye, as described in [70]. Absorbance was measured at λ = 620 nm (Bio Rad SmartSpec 3000). A standard curve with fixed concentrations of Evans blue was used to calculate dye extravasation into the brain. Data were expressed as mg of Evans Blue per g of brain tissue, as means ± standard deviation.

*Iron measurement*

Brain samples were dried (24 h, 99 °C), dissolved (3 M hydrochloric acid, HCl, 10% trichloroacetic acid; TCA; overnight at 65 °C) and diluted (10 µL) in water (590 µL). β-mercaptoethanol (10 µL), sodium acetate (pH 4.5; 500 µL) and bathophenanthroline-disulfonic acid (80 µL) were added to each sample (37 °C, 1 h). Absorbance was measured using a microplate reader (Bio-Rad 3550-UV) (λ = 535 nm).

*Isolation of brain-infiltrated immune cells*

Mice were sacrificed, perfused in toto (20 mL PBS) and brains were collected in HBSS, finely minced and digested in collagenase VIII (0.2 mg/mL, Sigma-Aldrich, Ref. No C2139; Darmstadt, Germany) for 30 min at 37 °C. Brains were homogenized by passing samples through a 100 µM strainer and collected in 20 mL HBSS. Next, samples were centrifuged for 10 min at 1500 rpm at 4 °C. Supernatants were discarded, and brains were washed in 20 mL HBSS and centrifuged again for 10 min at 1500 rpm at 4 °C. In order to separate the infiltrated leukocyte fraction, samples were resuspended in 10 mL of 30% Percoll gradient (GE Healthcare, Ref. No 17-0891-01; Darmstadt, Germany) and centrifuged for 20 min at 2500 rpm at room temperature, without break and acceleration. Myelin forming the upper layer was carefully removed, and brain-infiltrated leukocytes, precipitated at the bottom of the tube, were resuspended in 30 mL of PBS and centrifuged again for 10 min at 1500 rpm at 4 °C. The pellet obtained was then resuspended with 450 µL of PBS supplemented with 2% heat-inactivated FCS (Invitrogen) before being stained.

*Flow cytometry staining*

The total number of infiltrated immune cells was measured by flow cytometry using a known concentration of reference 10μm latex beads suspension (Polysciences Europe GmbH, Ref. No CC10N-10; Hirschberg an der Bergstrasse, Germany), co-acquired with a pre-established volume of cellular suspension. Dead cells were excluded by Propidium Iodide. Singlets were gated among live cells based on size and granularity. Cells were stained with Fc block (anti-CD16/CD32; BD Pharmingen™- Ref. No 553141; Madrid, Spain) to prevent non-specific binding for 20 min at 4 °C. Cells were then washed in PBS supplemented with 2% heat-inactivated FCS and stained as follows. The presence of resident microglia (CD11b+CD45int) and CNS infiltrates (CD45hi), such as Helper and Cytotoxic T cells (TCRβ+CD4+ and TCRβ+CD8+), was determined. Their activation was assessed by the expression of surface markers, such as CCR2 and MHC II for microglia or CD44 and CD62L for T cells. Different cell populations infiltrating the brain were determined by using antibodies directly conjugated to PE, PE/Cy7, PercepCy5.5, APC, FITC, BV421, BV510, and/or APC/Cy7 (purchased by BD Biosciences, Franklin Lakes, NJ, USA or eBioscience, San Diego, CA, USA). Cells were analyzed on a FACSCanto II (BD Biosciences) cytometer. FACS data were analyzed with FlowJo V10.

Antibodies were purchased by BioLegend (APC/Cy7 and BV421 anti-mouse CD45, Ref. No. 103115 and Ref. No. 103133; PE/Cy7 anti-mouse CD11b, Ref. No. 101215; PE and BV510 anti-mouse TCR β chain, Ref No. 109208 and Ref. No. 109233; APC/Cy7 anti-mouse CD8, Ref. No. 100714; Pacific Blue anti-mouse MHC II, Ref. No. 107620; APC anti-mouse CD44, Ref. No. 103012; PE/Cy7 anti-mouse CD62L, Ref. No. 10441; Amsterdam, The Netherlands), BD Biosciences (Pacific Blue anti-mouse CD4, Ref No. 558107; Madrid, Spain) and R&D SYSTEMS (PE anti-mouse CCR2, Ref. No. FAB5538P; Abingdon, UK).

*Calcein measurement*

Isolated cells were stained with Calcein AM Permeant Dye (ThermoFisher, Ref. No C1430; Bleiswijk, The Netherlands), which was added to the antibody mix and analyzed on a FACSCanto II (BD Biosciences) cytometer.

*qRT-PCR*

Total RNA was isolated from mouse brains using TRIzol (GRISP, Ref. No GB23.0100; Porto, Portugal) and the RNeasy Mini Kit (Machery-Nagel; Dueren, Germany), from blood using the NucleoSpin^®^ RNA Blood (Machery-Nagel; Dueren, Germany) and from cell suspension using the NucleoSpin^®^ RNA XS (Machery-Nagel, Ref. No 12733391; Dueren, Germany). Total RNA was retrotranscribed to cDNA (Transcriptor First Strand cDNA Synthesis Kit, ThermoFisher LTI, Ref. No 18080-051; Bleiswijk, The Netherlands) for PCR with Power SYBR Green PCR master mix (BioRad, Ref. No 1725124; Madrid, Spain). Transcript number was calculated from the threshold cycle (Ct) of each gene with a 2^−ΔΔCT^ method (relative number), normalized to ArbP0 or GADPH and expressed as fold induction of animals used as controls. PCR primers include: *Fth* Fwd: 5′-CCATCAACCGCCAGATCAAC -3′; *Fth* Rev: 5′- GCCACATCATCTCGGTCAAA -3′; *TH* Fwd: 5′- GGTATACGCCACGCTGAAGG -3′; *TH* Rev: 5′- TAGCCACAGTACCGTTCCAGA -3′; *ArbP0* Fwd: 5′- CTTTGGGCATCACCACGAA -3′; *ArbP0* Rev: 5′- GCTGGCTCCCACCTTGTCT -3′; *GADPH* Fwd: 5′- ACCACAGTCCATGCCATCAC -3′; *GADPH* Rev: 5′- CACCACCCTGTTGCTGTAGCC -3′.

*DNs isolation and culture*

DNs were isolated, following the protocol described in [71].

Briefly, mouse embryos were removed from the uterus of pregnant females at E12.5 and placed in ice-cold HBSS. The ventral midbrain was dissected at the microscope by separating the isthmus and mesencephalic-diencephalic boundary region with the aid of a curved scissor. The meninges were removed, and a cut in the mediodorsal midbrain was performed to flatten the tissue on a Petri dish. By using surgical blades, the wings of the butterfly shape, representing the substantia nigra, were dissected, and isolated regions were transferred into a falcon tube containing 15 mL of ice-cold HBSS. The samples were homogenized in 1 mL of pre-warmed solution of 0.05% trypsin-EDTA and incubated at 37 °C for 5 min. The enzyme activity of the trypsin-EDTA was stopped by adding 1 mL of DMEM/F12, supplemented with serum. The samples were centrifuged gently so as not to damage dissociated cells and washed in 1 mL of complete medium. This procedure was conducted twice, and with the aid of a fire-polished glass pipette, the tissue was triturated until obtaining a fine cell suspension. The samples were centrifuged at 400× *g* for 5 min at RT, and the medium was removed. Cells were then resuspended in 1 mL of complete medium, supplemented with N2. Once their viability was determined, through the Trypan Blue exclusion method, cells were seeded, on 24-well plates coated with Poly-L-ornithine/Laminin, in DMEM/F21, at a density of 150,000 cells per well. Plates were kept in a humidified tissue culture incubator and maintained at a 37 °C and 5% CO_2_. Experiments were performed 2 weeks after.

*Microglia isolation and culture*

Microglia were isolated as follows.

Mouse pups, at post-natal days 0–3, were decapitated to expose the skull and to dissect brain hemispheres. Brain samples were transferred into a falcon tube containing 30 mL of cold HBSS. Meninges were removed, and the brains were dissected into small pieces. Those were placed in a falcon tube containing 5 mL of 0.05% trypsin-EDTA, to which 750 μL of DNase I (10 mg/mL) were added. The digestion was carried out by maintaining samples at 37 °C for 15 min. After trypsin inactivation, samples were centrifuged at 400× *g*, for 5 min, at 20 °C. Cells were resuspended in 5 mL of DMEM/F12 supplemented with 10% FBS and 1% penicillin/streptomycin (10,000 units; Invitrogen, ThermoFisher Scientific, Waltham, MA, USA). Microglia were then seeded in a T75 culture flask, previously coated with poly-l-lysine solution (0.1 mg/mL in sterile water), containing 10 mL of culture medium and maintained at 37 °C and 5% CO_2_. On day 2, the macrophage colony-stimulating factor (M-CSF; 100 ng/mL) was added to the medium, along with and granulocyte-macrophage colony-stimulating factor (GM-CSF; 100 ng/mL). Microglia were cultured for 3 weeks, and the medium was changed every week. At confluency, cells were detached with 0.05% trypsin-EDTA and seeded into 24 well plates for experiments.

*Cytotoxicity Assay*

Cells were washed with PBS and exposed to the sera, collected from tested mice, i.e., aged animals or previously exposed to LPS or CLP, and pooled. Aliquots were prepared by diluting pools 1:10, and 50 μM of sera were added to the plate. All cells were covered with parafilm, allowing the sera to spread in the well and stimulate all the plates. In vitro, cytotoxicity was tested 24 h after. Cell death was also assessed in response to Fe treatment, provided as a ferric sulfate solution, Fe_2_(SO_4_)_3_ (100 μM), for 12 h. Cells were pre-treated with deferiprone (75 μM), 1 h before Fe_2_(SO_4_)_3_ and maintained thereafter. Control cells were washed with PBS and exposed to sera collected from control mice, i.e., young or non-treated animals. Viability was assessed by a crystal violet assay, as previously described [66,72,73].

*ELISA*

TNF and IL-6 were measured by ELISA according to manufacturer instructions (Thermo Fisher Waltham, MA, USA, 88-7324-22 and 88-7064-22, respectively).

*Statistical analysis*

Locomotor dysfunction or flow cytometry experiments were performed between 2 to 3 times, using between 2 and 4 mice per time. The results were pooled and expressed as mean ± standard deviation to assess statistical differences. The total number of mice per condition varied and is indicated in figure legends. In the graphs, each dot represents a mouse. ELISA and qRT-PCR experiments were conducted twice when the results reproduced the same trend. Representative graphs are shown and expressed as mean ± standard deviation.

Statistically significant differences between the two groups were assessed using a two-tailed unpaired Mann-Whitney test or *t*-test, according to data distribution. Normal distributions were confirmed using the Kolmogorov-Smirnov test. Statistical differences between groups following a non-normal distribution were assessed by applying the Mann-Whitney. Comparisons between more than two groups were carried out by one-way ANOVA. No statistical method was used to predetermine the sample size. All statistical analyses were performed using GraphPad Prism 9 software. Differences were considered statistically significant at a *p*-value < 0.05. NS: Not significant.

## 5. Conclusions

In this study, we demonstrated that pro-inflammatory priming of the brain sensitizes the development of PD. The sub-chronic inflammation that occurs during aging or immune system activation in response to the infection might increase the risk of PD. Peripheral factors play a crucial role in symptom onset, and our study suggests the possibility that early therapeutic interventions can protect the brain from this multifactorial disorder. Our work might also highlight the reasons for the inefficacy of drug testing in PD trials, which include already diagnosed cohorts. A possible explanation could be that disease progression is influenced by multiple events. Neurodegeneration is the final step in a series of processes that originate in the periphery and subsequently affect the brain. Thus, new therapies should contemplate the fact that drugs targeting PD might not need to enter the brain, which so far is the limiting factor. Targeting disease etiology rather than clinical manifestations might protect patients from developing severe PD.

## Data Availability

The data that support the findings of this study are available from the corresponding author upon reasonable request.

## Supplementary Materials

The supporting information can be downloaded: https://www.mdpi.com/article/10.3390/ijms24097949/s1.
