# Supplementary material for: Pro-Inflammatory Priming of the Brain: The Underlying Cause of Parkinson’s Disease
Source: Int J Mol Sci. 2023 Apr 27;24(9):7949. doi: 10.3390/ijms24097949 (PMC10178666; doi:10.3390/ijms24097949)
Supplement: Supplementary file 1 [file ijms-24-07949-s001.zip › ijms-2338779-supplementary.pdf]

Supplementary Figure S1

An increased number of activated microglia was found in aged PD mice, as quantified by flow cytometry through the expression of MHCII and CCR2. Higher counts and ac-tivation of Helper CD4 and Cytotoxic CD8 T cells were also observed in old animals, exposed to MPTP. These data reveal an exacerbated phenotype in PD-induced old an-imals, when compared to young MPTP-induced mice or age-matched controls.

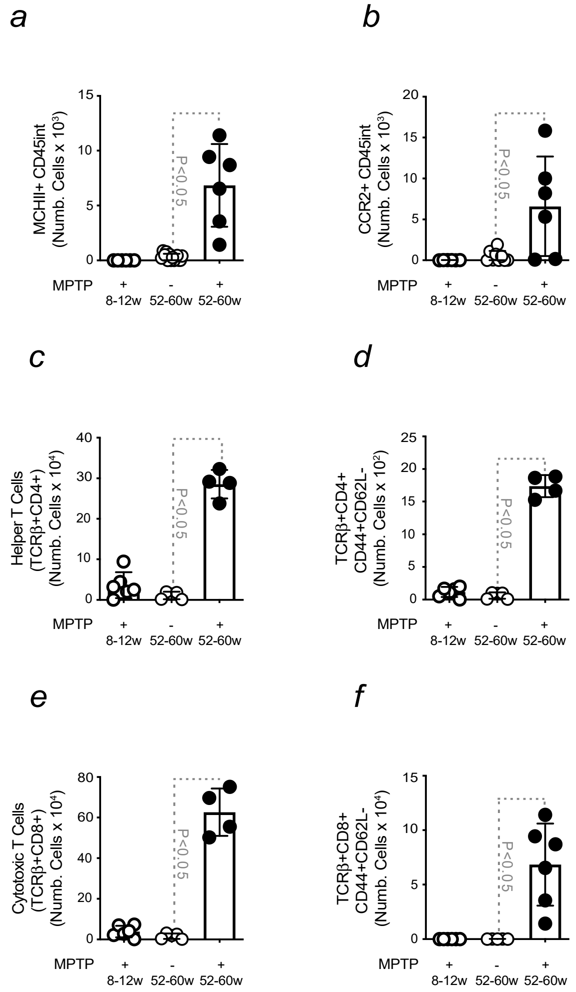

Figure S1. Aging increases the severity of Parkinson's disease. Number of activated microglia, by the expression marker (a) MHCII or (b) CCR2, measured by flow cytometry in young and old mice exposed or not to MPTP (15mg/kg, i.p., 3 injections, 2h apart). (c) Number and (d) activation of Helper CD4 T cells of mice as in a). (e) Number and (f) activation of Cytotoxic CD8 T cells of mice as in a). The results were expressed as mean ◎ SD (n=4-10 mice per group). Statistical analysis was performed by applying the one-way ANOVA test.

Supplementary Figure S2

An increased expression of the adhesion molecules VCAM and ICAM, measured by flow cytometry, were observed in old mice, when compared to young animals.

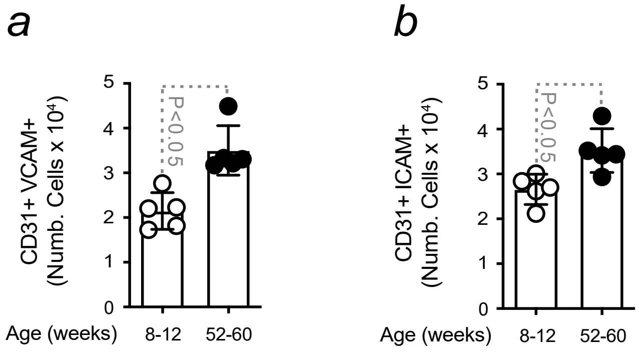

Figure S2. Aging-associated inflammation promotes BBB disruption. Number of isolated BBB cells expressing (a) VCAM or (b) ICAM, measured by flow cytometry in young and old mice. The results were expressed as mean ⊚ SD (n=5mice per group). Statistical analysis was performed by applying the one-way ANOVA test.

Supplementary Figure S3

Increased expression of the adhesion molecule VCAM, measured by qRT-PCR, was found in mice subjected to CLP, in relation to mice exposed to a sham surgery. This fa-vored the infiltration of Helper CD4 and Cytotoxic CD8 T cells, from the periphery to the brain of CLP-induced mice, upon MPTP induction, an effect that led to microglia activation and the appearance of a neuroinflammatory phenotype.

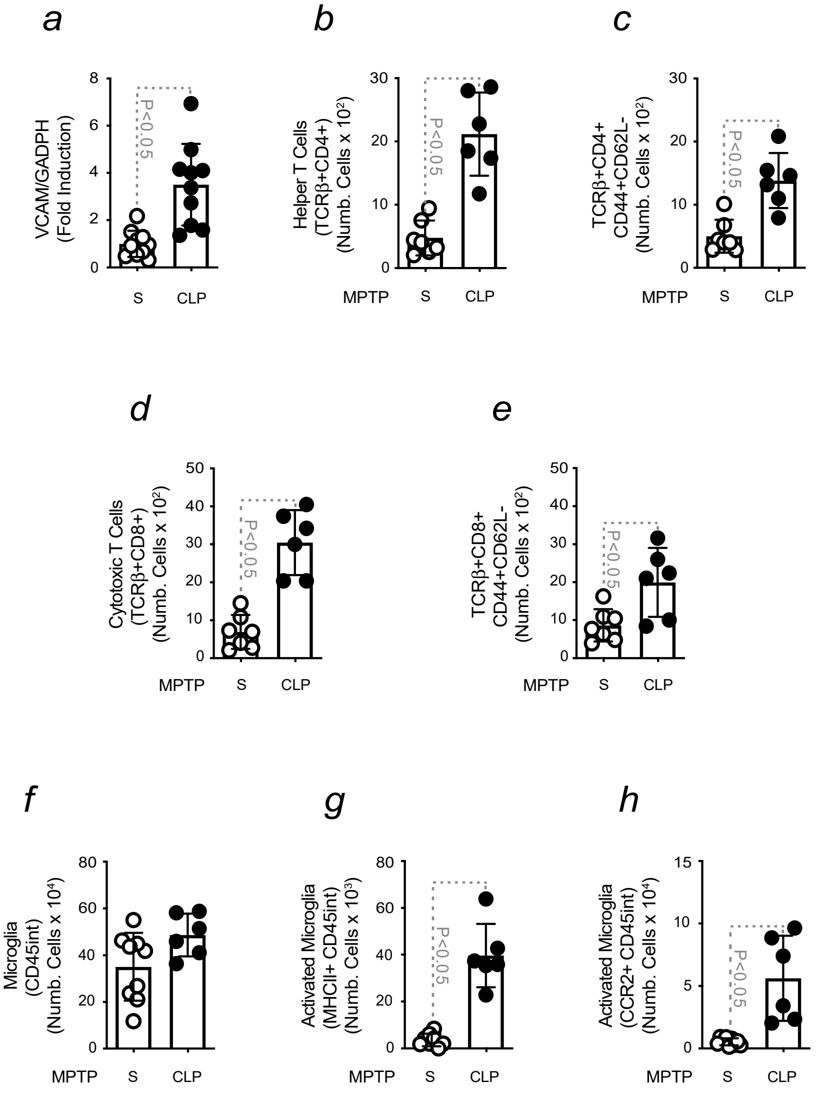

Figure S3. CLP-Infection-driven pro-inflammatory priming to the brain promotes neuroin-flammation. (a) Expression of VCAM, normalized to GADPH and measured by qRT-PCR in mice subjected to CLP or Sham (S) surgery. The results were expressed as mean ± SD (n=10 mice per group). The Student's t-test was applied to define statistical differences. (b) Number and (c) acti-vation of Helper CD4 T cells of mice as in a). (d) Number and (e) activation of Cytotoxic CD8 T cells of mice as in a). (f) Number and activation of microglia, measured by flow cytometry, through the expression of activation markers (g) MHCII or (h) CCR2 of mice as in a). The results were expressed as mean ◎ SD (n=4-10 mice per group). Statistical analysis was performed by applying the one-way ANOVA test.

Supplementary Figure S4

Time course of CLP induction in mice, to assess the time of occurrence of peripheral immune cells infiltration into the brain and microglia activation, considered as an early manifestation of the neuroinflammatory phenotype observed.

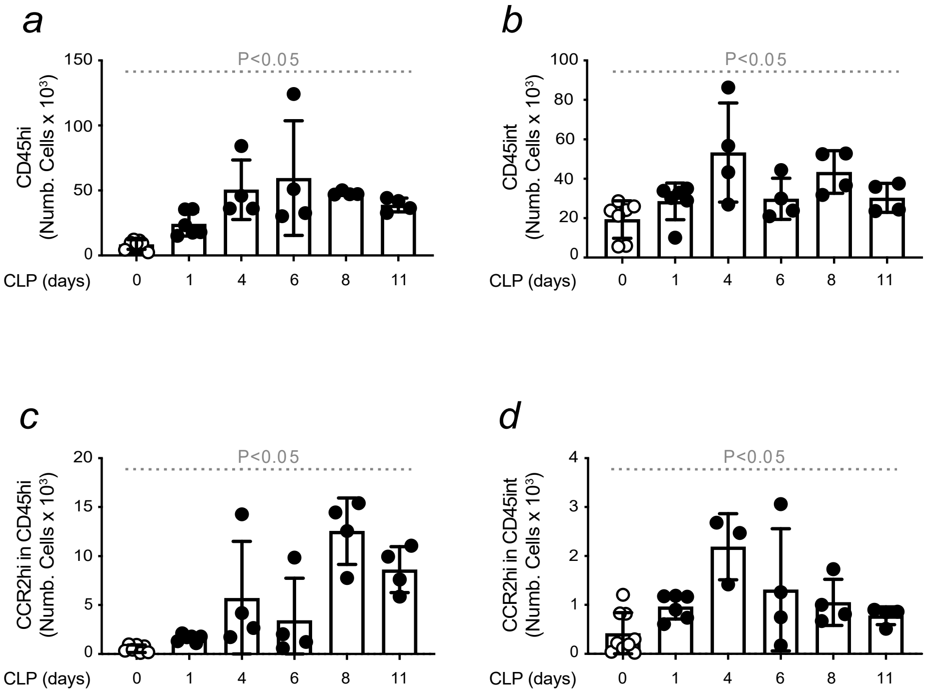

Figure S4. Time course of CLP-Infection-driven pro-inflammatory priming to the brain. Number of brain infiltrated leukocytes (a) and microglia (b) in mice subjected to CLP, during indicated post-infection days (c) activation of brain infiltrated leukocytes and (d) microglia, measured by flow cytometry, through the expression of activation marker CCR2, of mice as in a). The results were expressed as mean ⊚ SD (n=4-10 mice per group). Statistical analysis was performed by applying the one-way ANOVA test.

Supplementary Figure S5

Increased expression of the adhesion molecule VCAM, measured by qRT-PCR, was found in mice subjected to LPS, in relation to control mice. This favored the infiltration of Helper CD4 and Cytotoxic CD8 T cells, from the periphery to the brain of LPS-induced mice, upon MPTP induction, an effect that led to microglia activation and the appearance of a neuroinflammatory phenotype.

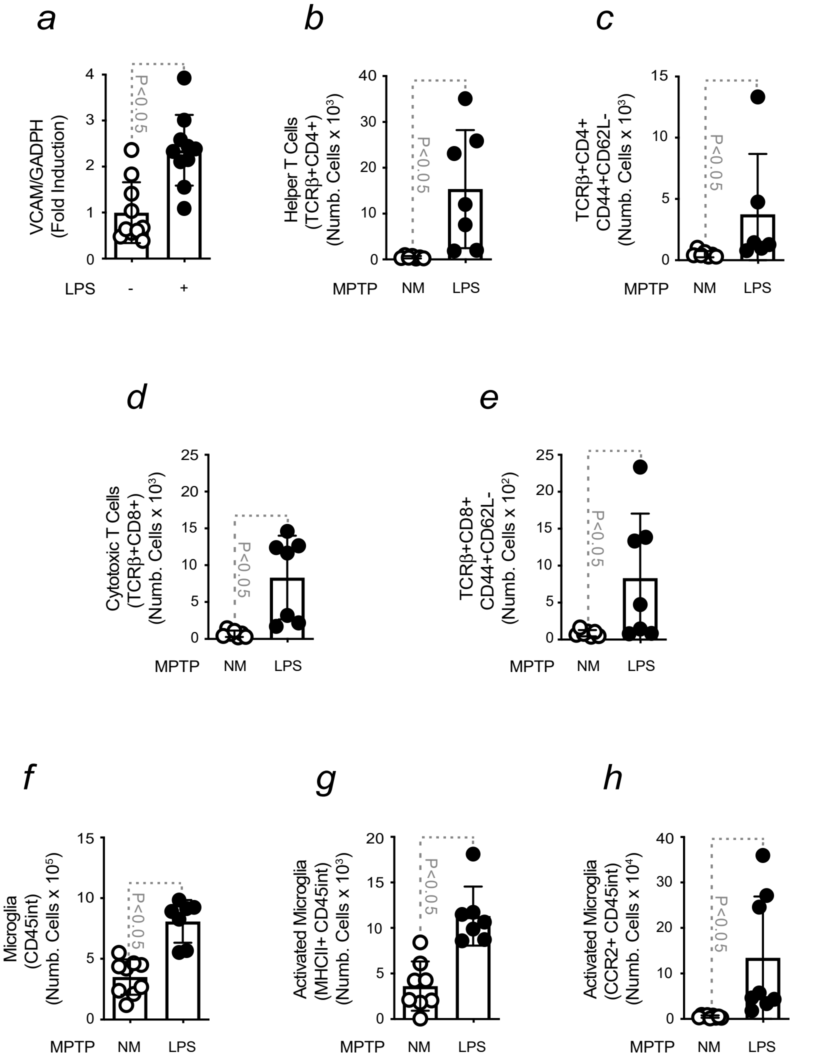

Figure S5. LPS-Infection-driven pro-inflammatory priming to the brain promotes neuroinflammation. (a) Expression of VCAM, normalized to GADPH and measured by qRT-PCR in mice induced or not with LPS. The results were expressed as mean ± SD (n=10 mice per group). The Student's t-test was applied to define statistical differences. (b) Number and (c) activation of Helper CD4 T cells of mice as in a). (d) Number and (e) activation of Cytotoxic CD8 T cells of mice as in a). (f) Number and activation of microglia, measured by flow cytometry, through the expression of activation markers (g) MHCII or (h) CCR2 of mice as in a). The results were expressed as mean ⊚ SD (n=4-10 mice per group). Statistical analysis was performed by applying the one-way ANOVA test.

Supplementary Figure S6

An increased infiltration of activated Helper CD4 and Cytotoxic CD8 T cells, from the periphery to the brain, was observed in CLP-induced mice. This was reduced by thera-peutically treating those animals with deferiprone. The same beneficial effect was also detected in relation to microglia activation. Hence, deferiprone diminished the neu-roinflammatory phenotype associated with the CLP-pro-inflammatory priming to the brain.

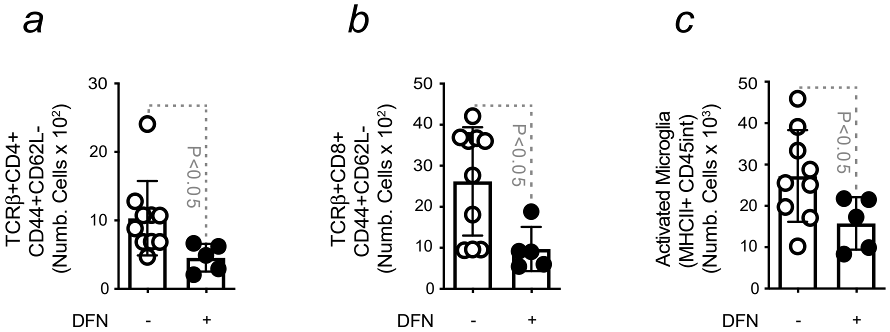

Figure S6. Deferiprone diminishes the neuroinflammation caused by CLP-driven pro-inflammatory priming to the brain. (a) Activation of Helper CD4 T cells, (b) Cytotoxic CD8 T cells and (c) microglia in CLP-induced mice therapeutically treated or not with deferiprone (DFN). The treatment lasted for 15 days, starting 5 days after CLP. The activation was measured by flow cytometry, through the expression of activation markers, CD44 and MHCII, for T cells and micro-glia respectively. The results were expressed as mean ◎ SD (n=5-10 mice per group). Statistical analysis was performed by applying the one-way ANOVA test.
